# Leukemia Associated Antigens: Their Dual Role as Biomarkers and Immunotherapeutic Targets for Acute Myeloid Leukemia

**Published:** 2007-02-14

**Authors:** Barbara-ann Guinn, Azim Mohamedali, Ken I. Mills, Barbara Czepulkowski, Michael Schmitt, Jochen Greiner

**Affiliations:** 1 Department of Haematological Medicine, King’s College London School of Medicine, The Rayne Institute, 123 Coldharbour Lane, London, SE5 9NU; 2 Department of Haematology, University Hospital of Wales, Heath Park, Cardiff, CF4 4XN, U.K; 3 Third Clinic for Internal Medicine, University of Ulm, Germany

**Keywords:** Acute myeloid leukemia, leukemia/tumor-associated antigens, immunotherapy, cancer-testis antigens, SEREX, microarray, mass spectrometry

## Abstract

Leukemia associated antigens (LAAs) are being increasingly identified by methods such as cytotoxic T-lymphocyte (CTL) cloning, serological analysis of recombinant cDNA expression libraries (SEREX) and mass spectrometry (MS). In additional, large scale screening techniques such as microarray, single nucleotide polymorphisms (SNPs), serial analysis of gene expression (SAGE) and 2-dimensional gel electrophoresis (2-DE) have expanded our understanding of the role that tumor antigens play in the biological processes which are perturbed in acute myeloid leukemia (AML). It has become increasingly apparent that these antigens play a dual role, not only as targets for immunotherapy, but also as biomarkers of disease state, stage, response to treatment and survival. We need biomarkers to enable the identification of the patients who are most likely to benefit from specific treatments (conventional and/or novel) and to help clinicians and scientists improve clinical end points and treatment design. Here we describe the LAAs identified in AML, to date, which have already been shown to play a dual role as biomarkers of AML disease.

## Introduction

**Figure 1:**
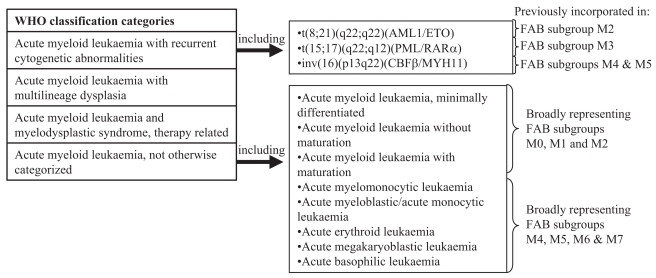
Subclassification of AML by the WHO system. Diagnosis of AML based on the World Health Organisation (WHO) classification system (based on review by Vardimann et al. 2002) which superseded the French-American-British (FAB) classification system.

Acute myeloid leukemia (AML) describes a heterogeneous group of diseases which are typified by the outgrowth of immature hematopoietic cells, known as blasts, in the bone-marrow and peripheral blood. Most patients with AML, who are younger than 60 years of age, achieve complete remission after polychemotherapy and/or stem cell transplantation (SCT) (Tallman et al. 2005). However, it is subsequent relapse with it’s high associated morbidity that leads to five year survival rates of approximately 50% (Lowenberg et al. 1999). It should be noted that the median age of AML patients is 63 years and for the majority of patients with AML, who are over 60 and ineligible for SCT, the rate of complete remissions are markedly reduced to 11% (Rowe et al. 2004). The karyotype provides the most important prognostic information in adult AML (Grimwade et al. 1998). Multivariate analysis revealed that an age above 70 was the other major negative prognostic factor for overall survival besides high-risk cytogenetics (Frohling et al. 2006). However, using conventional chromosome banding analysis, approximately 50% of AML patients lack chromosomal aberrations. For these AML patients, molecular genetic approaches become of major importance. The World Health Organization (WHO) classification system (Vardiman et al. 2002) (overviewed in Figure 1) replaces the preceding French-American-British (FAB) classification system and in addition to clinical data, also takes into consideration biological characteristics, such as morphology, cytochemistry, immunophenotype, cytogenetics and molecular biology. The separation of subgroups also allows the distinction of prognostic parameters and the identification of patients who are better suited to specific treatment strategies.

Immunotherapy offers an opportunity to remove residual diseased cells in remission and can potentially reduce or eliminate the risk of relapse. During the last 20 years significant improvements have occurred in our ability to identify tumor antigens. Originally leukemia associated antigens (LAAs) were identified by virtue of their role in leukemogenesis and were subsequently shown to be immunogenic. The serological analysis of recombinant cDNA expression libraries (SEREX) technique allowed the identification of LAAs due to their recognition by humoral responses but also led to the demonstration that these antigens were recognized by CD4^+^ and CD8^+^ T cells as well as B-cells. More recently microarray, single nucleotide polymorphisms (SNPs) and 2-dimensional gel electrophoresis (2-DE) have allowed the large scale analysis of gene, RNA and protein differences between patient and normal donor samples.

We have had a particular interest in identifying LAAs which may act as targets for the immunotherapy of AML. Tumor antigens such as Wilm’s tumor (WT1) (Menssen et al. 1995), preferentially expressed antigen of melanoma (PRAME) (Ikeda et al. 1997), proteinase 3 (PRTN3) (Molldrem et al. 1996) and the receptor for hyaluronic acid mediated motility (RHAMM/CD168) (Greiner et al. 2002) have been used with some success in phase I/II clinical trials (Greiner et al. 2005; Guinn et al. 2006b; Heslop et al. 2003; Li et al. 2006; Mailander et al. 2004; Oka et al. 2004) (reviewed elsewhere in Guinn et al. 2006b; Heslop et al. 2003). In addition, a growing group of LAAs have been shown to play a dual role as biomarkers in AML. In this review we describe some of these LAAs, whose capacity to act as biomarkers of disease stage, disease progression, relapse and survival have already been demonstrated.

## Associations between LAAs, Subgroups and Cytogenetics

Apart from acute promyelocytic leukemia (APL), none of the remaining subgroups of AML have a single cytogenetic aberration associated with all samples from that subtype (Figure 1). The PML-RARα oncogene is a chimeric protein produced by the t(15;17) translocation in APL and has been used as a target for DNA vaccinations in mice (Padua et al. 2003). This work clearly showed that a combination of conventional treatment (in this case all-trans-retinoic-acid) and immunotherapy could effectively improve survival rates in an animal model of APL. This work is now progressing into human clinical trials. Of note secondary cytogenetic changes occur in 32% (of 161) APL patients examined and are associated with longer complete remission and event free survival (P = 0.03) in patients treated with chemotherapy (Slack et al. 1997).

However, some genes are overexpressed in AML and are associated with specific subgroups of AML, such as PRAME. PRAME was first identified as an antigen in human melanoma by virtue of patient CTLs (Ikeda et al. 1997). PRAME is expressed in 45% of AML M2 and 75% of AML M3 patients (Matsushita et al. 2004) and PRAME positive patient samples have been shown to be susceptible to lysis by PRAME-specific CTLs (Morandi et al. 2006).

Heat shock proteins (HSPs) may be referred to as molecular chaperones and are found in both normal and cancerous cells. These proteins are immunogenic, with the specificity of the immune response being dictated by the peptides they chaperone (reviewed in Binder, 2006). Recent studies have investigated their frequency of expression and shown that HSP90 and 110 expression are associated with FAB M5 subtype and unfavorable and intermediate karyotypic groupings (Thomas et al. 2005).

WT1 is overexpressed in most types of human adult leukemia, and overexpressed in 90% of AML patients, except in AML FAB group M5, where expression occurs less frequently (Bergmann et al. 1997; Inoue et al. 1994; Miwa et al. 1992). A phase I clinical trial was recently described in which AML, MDS, breast and lung cancer patients were injected with either a naturally occurring or modified WT1 peptides. Twelve of 20 patients (who could be assessed) showed clinical responses and these could be clearly correlated with increased frequencies of the number of WT1 specific CTLs (Oka et al. 2004).

RAS is one of the most frequently occurring mutations in cancer and has been shown to play roles in the subversion of proliferation and differentiation. RAS has also been shown to lead to enhanced apoptosis in preleukemic bone marrow (Darley et al. 1997) and conversely growth factor independence, leading to the avoidance of apoptosis in immortalized cells (Kinoshita et al. 1995). Peptide immunizations with a 13’mer peptide which represented patients own tumor ras led to ras-specific CD4^+^ and/or CD8^+^ responses. In addition, CD8^+^ T cells specific for the Gly to Val mutation led to the killing of cells from a HLA-A2 matched tumor cell line carrying the corresponding mutation, but not those cells carrying wild type RAS (Khleif et al. 1999). The NRAS mutation has been shown to be overrepresented in AML patients with the t(3;5) translocation and under represented in the t(15;17) subgroup (P < 0.001 for each) (Bowen et al. 2005). In the same study KRAS was shown to be over-represented in the inv(16) group (P = 0.004) while RAS mutations and FLT3 ITD were rarely found to co-exist (P < 0.001).

Bcl-2 is an anti-apoptotic protein which plays a pivotal role in preventing apoptotic cell death. Similarly it is thought that the targeting of cells which overexpress Bcl-2 has the potential to kill only those cancer cells with an associated growth advantage. Andersen et al. (Andersen et al. 2005) demonstrated that patients with AML had spontaneously arising CTL-reactivity against Bcl-2 in patients but not normal donors, suggesting that vaccines against Bcl-2 could be effective against tumor cells which overexpress this protein. Bcl-2 overexpression has been shown to be associated with the M4 and M5 subtypes (p < 0.01) (Daneshbod et al. 2005).

Telomerase reverse transcriptase (hTERT) is involved in telomere elongation and is frequently found to be expressed in human cancers and rarely in normal tissues. It has been the target of a number of immunotherapy clinical trials (reviewed in Carpenter and Vonderheide, 2006), due to it’s tumor associated expression. Monocyte derived dendritic cells from an AML cell line, were used to generate functional CTL clones with specificity for hTERT (Santegoets et al. 2006). In addition to it’s expression in cancer and cancer stem cells, in AML hTERT levels have been found to be associated with CD34 expression and chromosomal abnormalities (P = 0.01 and P = 0.001, respectively) (Xu et al. 1998). Elevated hTERT levels have been associated with patients less likely to achieve complete remission (Zhang et al. 1996) and in patients with relapsed, rather than those newly diagnosed with AML (Huh et al. 2005; Tatematsu et al. 1996).

Using microarray analysis on 195 AML patient samples at presentation Guinn et al. (Guinn et al. 2006a) showed an association between the expression of the LAA, meningioma antigen 6 (MGEA6) (Heckel et al. 1997), and FAB subgroup. The renal antigen 1 (RAGE-1) (Gaugler et al. 1996) was also found to segregate with the less differentiated forms of AML as dictated by FAB subgroup but not by WHO subgroup, suggesting that both RAGE-1 and MGEA6 could be targeted, by immunotherapy, to the less differentiated forms of AML as indicated by FAB subtype.

Greiner et al. (Greiner et al. 2006) also examined a large dataset of 116 AML patient samples which had previously been analyzed by microarray. They showed an association between PRAME, PRTN3, LAMR1 and G250/CA9 and distinct karyotypes. Higher levels of PRAME expression was associated with t(8;21), del(7q)/–7 and t(15;17) while complex karyotypes and inv(16) were associated with lower levels of PRAME expression. A higher relative level of PRTN3 was associated t(8;21) and inv(16) while t(9;11) and del(7q)/–7 were associated with lower levels. Higher expression of LAMR1 and G250/CA9 was associated with del(7q)/–7, while G250/CA9 higher levels were associated with t(9;11). Complex karyotypes and t(15;17) were associated with lower relative levels of expression of G250. They also found WT1 expression to be associated with the presence of FLT3 ITDs.

## Tumor Antigens as Biomarkers of Survival

Bergmann et al. showed that high levels of WT1 mRNA in AML were associated with poor long term outcome (Bergmann et al. 1997; Trka et al. 2002) while others have found no correlation (Gaiger et al. 1998; Greiner et al. 2006; Schmid et al. 1997; Yanada et al. 2004). This may be explained in part by the cut off for “high levels” of expression chosen by the authors, the methods of detection and the sample source. Quantitative assessment of WT1 transcripts using a nested reverse transcription PCR titration assay indicated an association between the good and standard cytogenetic risk groups and poorer outcome in >90% of patients (Garg et al. 2003). The same study also showed a positive correlation between WT1 transcript levels and remission rates, disease free survival and overall survival (P = 0.003). Wt1 has been shown to regulate the expression of the proto-oncogene bcl-2, and expression of the two genes correlate significantly (Karakas et al. 2002). In patients <60 years of age, expression of BCL-2 and WT1 were associated with a reduced rate in continuing complete remission and increased death rate, in contrast to patients >60 years, where expression of these genes had no association with survival rates. Of note RAS mutations were not found to influence clinical outcome in terms of overall survival, disease free survival, complete remission or relapse rates (Bowen et al. 2005).

In addition to it’s role as a marker of prognosis for AML patients (Kottaridis et al. 2001; Kottaridis et al. 2002) FLT3 has been used as a target for a humanized antibody (Li et al. 2004) which has been shown to have anti-FLT3 effects on leukemic cell lines in vitro and in xenograft models. Other groups have examined FLT3-ITD expression in AML and although it has been found to be associated with AML progression from MDS and a worse outcome in patients with AML in general (Pollard et al. 2006; Shih et al. 2004), this is still a matter for debate in APL. Gale et al. (Gale et al. 2005) examined FLT3 mutations in 203 PML-RARα positive APL and looked for associations between biological characteristics and response to targeted therapy. They found that patients with mutant FLT3 had a higher rate of induction death (P = 0.04) but there was no significant difference in relapse risk or overall survival at 5 years. Callens et al. (Callens et al. 2005) found that FLT3-ITD was associated with high white cell count, high Sanz index and M3 variant isoforms. Although FLT3-ITD was associated with a trend towards poorer overall survival, RAS and FLT3-ITD mutations were not associated with complete remission, induction death or death in complete remission.

In 2002, Steinbach et al. (Steinbach et al. 2002) showed that the PRAME gene was expressed by CD34^+^ stem cells which they suggested may constitute a problem for its targeting in tumor immunotherapy. They found overexpression of PRAME in 62% (n = 31) of childhood AML patients and that the rates of overall and disease-free survival were higher in patients with elevated levels of expression. They also found that PRAME expression was significantly higher in patients with t(8;21). Conversely, in adult AML Paydas et al. (Paydas et al. 2005) found that 30% of AML patient samples analyzed had PRAME expression (n = 74) and did not find any correlation between PRAME expression and clinical characteristics, including response to therapy, progression-free and overall survival. PRAME was reported to have its highest level of expression in patients harboring the t(8;21) translocation and that unlike WT1, its expression levels correlated inversely with prognosis (Steinbach et al. 2002).

Bcl-2 is one of the antiapoptotic, and bax one of the proapoptotic genes, which regulate the mito-chondrial-mediated pathway of apoptosis (Oltvai et al. 1993). Bcl-2 has been shown to prolong the survival of leukemia cells (Mariano et al. 1992). Del Poeta et al. (Del Poeta et al. 2003) showed that an elevated bax/bcl-2 ratio was associated with a longer overall survival and disease free survival in patients (p = 00001 and p = 0.019, respectively) in a study of 225 de novo AML patients by flow cytometry. Of particular interest bax/bcl-2 levels accurately predicted clinical response and outcome in patients with normal or unknown cytogenetics. In this group of patients (n = 147, 65% of the total patients analyzed) a higher bax/bcl-2 ratio was associated with a higher complete remission rate (p = 0.0016) and a longer overall survival. These data suggest that the measurement of the bcl-2:bax ratio in patient samples may be used as a sensitive indicator of clinical outcome. Previously Borg et al. (Borg et al. 1998) had shown that bcl-2 expression was not related to survival while Ong et al. (Ong et al. 2000) had shown that high bax expression was a good prognostic indicator in AML, with patients exhibiting high bax expression at diagnosis having a significantly better prognosis for disease-free, event-free and overall survival (p = 0.016). This suggests that it is in fact bax which is the prognostic indicator in the studies of bax/bcl-2 levels. However earlier studies indicated a poor prognostic association with high bcl-2 expression in AML (Russell et al. 1995) and with shorter survival, complete remission and complete remission durations (Daneshbod et al. 2005), indicating that either low bax and/or high bcl-2 levels give a worse prognostic indication. Survivin is a member of the inhibitor of apoptosis protein family, which by immunocytochemistry was never found in normal samples but was found in almost all AML patient samples examined (Invernizzi et al. 2004). Wagner et al. (Wagner et al. 2006) examined Survivin, the predominant transcript variant in AML, for it’s expression and that of the splice variants survivin-2B and survivin-ΔEx3 in 74 adults with AML. They found that low expression of survivin-2B correlated with better overall survival and event free survival (p ≤ 0.01; 27 months vs. 10 months) unlike Survivin and survivin-ΔEx3.

In a study of 98 newly diagnosed AML patients, cytogenetics, CD34 positive expression, multidrug resistance positive expression and HSP110 positive expression were found to be major prognostic factors for overall survival (Thomas et al. 2005). The group found that despite treatment differences higher expression of all HSPs seemed to correlate with lower complete remission rates and shorter survival. In addition they found that expression of all HSPs were related to bcl-2 expression perhaps reflecting the already established role of HSPs (such as HSP27, 60, 70 and 90) in apoptotic pathways.

By microarray analysis Guinn et al. (Guinn et al. 2006a) found no association between RAGE-1 and/or MGEA6 expression (present or absent calls) and survival, however, MGEA6 expression was found to occur more frequently in patients who had cytogenetic abnormalities associated with poor versus standard versus good survival and the difference between each was statistically significant (p < 0.0001, Chi2 pairwise analysis). Of note, no correlation was found between present and absent calls and patient survival when examining *CA9*, *PRAME*, *RHAMM*, *RAGE-1* or *MGEA6* expression individually or in combinations of *CA9*, *PRAME* and *RHAMM* or *RAGE-1* and *MGEA6* (Guinn et al. 2007). However when high or low MGEA6 expression (as compared to the median level of expression) was examined in patients with present calls, a trend towards improved survival and elevated MGEA6 expression (p = 0.148) was found.

Greiner et al. (Greiner et al. 2006) found a correlation between high G250/CA9 mRNA expression levels (as compared with the median in G250/ CA9 expressers) and a longer overall survival (p = 0.022). This trend was replicated with PRAME (p = 0.103) and a similar suggestion was found for RHAMM (p = 0.284). Of interest they showed that expression of at least one of the three TAAs, RHAMM/HMMR, PRAME or G250/CA9, provided the most favorable prognostic score (P = 0.005). They also found no correlation between PRTN3, WT1, TERT or LAMR1 and outcome, however elevated levels of BCL-2 suggested poorer overall survival although this was not statistically significant (p = 0.250).

Rather than using the median as a cut off point above which G250/CA9, PRAME, RHAMM and MGEA6 expression were shown to be associated with improved prognosis (Greiner et al. 2006), there has been a recent suggestion that a group of “distinctly” high expressing AML patients could be identified by real-time PCR (RQ-PCR) (Guinn et al. 2007). In the case of CA9/G250 and PRAME the existence of a group of patients (36% and 54%, respectively) with a 1 log higher expression than that of normal donors and the remaining AML patients were found. It was suggested that survival in this group of distinctly high expressers should be compared to normal donors and the remaining AML patients for future analyses of survival.

## Biomarkers for Minimal Residual Disease (MRD)

The analysis of MRD provides an indication of when molecular remission is achieved, through the RQ-PCR analysis of transcript levels from genes which are associated with disease load. However until recently AML has lacked good markers of MRD (reviewed in Raanani and Ben-Bassat, 2004). Data from studies using RQ-PCR protocols to monitor MRD in AML patients with t(8;21), inv(16) and t(15;17) transcripts (Krauter et al. 1999; Marcucci et al. 2001; Martinelli et al. 2003; Miyamoto et al. 1995; Tobal et al. 2001; Tobal et al. 2000; Tobal and Yin, 1996; Viehmann et al. 2003) support the capacity of RQ-PCR analysis to be used to detect very low transcript levels and their rise with ensuing relapse (reviewed in Guinn and Tobal, In press; Raanani and Ben-Bassat, 2004; Yin & Grimwade, 2002). However, to date, AML patients with identified fusion genes represent approximately 55% of all AML cases and encompass a vast list of aberrations. There is, therefore, a need to identify alternative gene targets that are either specifically expressed or significantly upregulated in leukemic cells in the majority of AML patients. The exception to this is PML-RARα in the AML M3 subgroup which provides an excellent and very specific marker for MRD. Studies have shown that rigorous testing of PML-RARα transcripts and pre-emptive treatment at the point of molecular relapse can improve survival rates in a relatively small group of low risk patients (reviewed in Grimwade and Lo Coco, 2002).

We would expect that LAAs such as RHAMM, PRAME, and WT1 (reviewed in (Sugiyama, 1998) would provide better markers of MRD due to their frequent expression in AML patients samples as compared to cancer-testis (CT) antigens such as HAGE (Adams et al. 2002), BAGE (Greiner et al. 2004), RAGE-1 (Guinn et al. 2005b), PASD1 (Guinn et al. 2005a) and MAGE-A3 (Martinez et al. 2007) whose expression was, at most, found in 33% of AML patients at presentation. Although CT antigens show leukemia specific expression and are not detected in normal tissues, LAAs are often found to have elevated levels of expression in leukemia cells compared with equivalent normal donor cells. Greiner et al. (Greiner et al. 2004) reported the detection of high expression levels of a number of LAAs including MPP11, RHAMM, WT1, PRAME, G250, hTERT, and BAGE using RQ-PCR in AML patients. In addition they reported that RQ-PCR showed a tumor-specific expression of the antigens BAGE, G250 and hTERT, as well as highly tumor-restricted expression for RHAMM, PRAME and WT1. Antigens such as WT1 and PRAME have already been shown to play a predictive role in the monitoring of MRD (Greiner et al. 2006; Inoue et al. 1994; Matsushita et al. 2001).

WT1 has been shown to be a marker of MRD by several groups (Cilloni et al. 2002; Inoue et al. 1996; Osborne et al. 2005; Weisser et al. 2005) reviewed in (Sugiyama, 1998), with the suggestion that this would provide a tool for monitoring MRD in 70% of AML patients (Ostergaard et al. 2004). Cilloni et al. (Cilloni et al. 2002) showed that normal and regenerating bone marrow samples and purified CD34^+^ cells expressed minimal amounts of the WT1 transcripts. They also showed that WT1 transcripts were frequently undetectable in normal peripheral blood. In contrast they found high levels of WT1 in the peripheral blood and bone marrow samples from acute leukemia patients at diagnosis and that the WT1 transcript levels followed the pattern of other molecular markers, such as fusions transcripts, used in current MRD monitoring protocols. Most significantly they and Barragan et al. (Barragan et al. 2004) found that increased WT1 transcript levels in the bone marrow and/or peripheral blood were predictive of impending relapse. Garg et al. (Garg et al. 2003) were able to show that the monitoring of WT1 transcripts in AML patients by RQ-PCR could predict relapse in patients up to 6 months before the onset of clinical relapse. The data showed that *WT1* transcript levels at various phases of the disease (presentation, post induction, post consolidation chemotherapy) could add important prognostic information in distinguishing patients who have a poor prognosis from those who would respond well to chemotherapy and achieve long term remission (Garg et al. 2003). In slight contrast, Weisser et al. (Weisser et al. 2005) found that although WT1 levels correlated with a shorter overall survival and event free survival at days 61–120 and 121–180, after the start of chemotherapy, they did not correlate with shorter overall survival and event free survival at diagnosis or between days 16–60.

PRAME, which is expressed in 40% of adult AML patients (Tajeddine et al. 2006), has been shown to be a useful marker for MRD particularly where other tumor specific markers are unavailable (Matsushita et al. 2001; Matsushita et al. 2003). Tajeddine et al. (Tajeddine et al. 2006) found that PRAME expression closely correlated with AML1/ ETO levels in AML patients harboring the t(8;21) translocation. In addition they found that PRAME expression highly correlated with clinical data when sequentially following AML patients from onset to cytological remission or relapse.

## Assays for Identifying New Biomarkers/LAAs

Many of the earliest LAAs were identified as products of cytogenetic rearrangements and later targeted as disease specific antigens. Padua et al. (Padua et al. 2003) have described their work targeting PML-RARα in AML in mouse studies which are now progressing to clinical trials (reviewed in (Robin et al. 2005). Other ways in which LAAs have been identified, have been through the identification of genes which play a role in leukemogenesis and were later shown to be targets for immunotherapy, through reverse or conventional immunology (reviewed in Guinn et al. 2006b).

We and others have used the SEREX technique (Sahin et al. 1995) to identify a large number of tumor antigens in presentation AML samples (Chen et al. 2005; Greiner et al. 2000; Greiner et al. 2003; Greiner et al. 2002; Guinn et al. 2005a). Some of these antigens had already been shown to play a role in the pathogenesis of solid tumors (such as NAB2, NY-BR-1 and ACVR2) or in leukemogenesis (including MYH11*,* RHAMM, MAPK10, BRAP and TRIP11). In addition these studies identified known leukemia antigens such as RHAMM, MAGE-A1 as well as novel targets for leukemia immunotherapy such as PASD1, SSX2IP and BAGE.

Microarray analysis of gene expression has provided a powerful tool to characterize the molecular mechanisms underlying many cancers. With regards to AML, cDNA microarray analysis has allowed the identification of genes that are differentially expressed in leukemic blasts as compared to normal hematopoietic lineages, indicate prognosis, show the gene expression effects of varying treatment regimes and identify the genes involved in the processes underlying disease (reviewed in Bullinger and Valk, 2005; Grimwade and Haferlach, 2004). cDNA microarray has been used to differentiate cytogenetic groups and to identify new subgroups within the intermediate risk group with a normal karyotype (Bullinger et al. 2004). More recently microarray was used to identify LAAs which were expressed in AML cells, but not equivalent normal donor cells (Guinn et al. 2005b), and Greiner et al. (Greiner et al. 2006) used microarray to show associations between G250/CA9, PRAME and RHAMM expression and survival. In addition Chapiro et al. (Chapiro et al. 2006) used microarray to show that AML M3 has several features of a T-lymphoid cell changing our understanding of the molecular mechanisms underlying this unique subgroup of AML. The limitation of transcriptomics, is that transcript levels do not necessarily correlate with protein expression and it cannot detect important post-translational modifications. Balkhi et al. (Balkhi et al. 2006) recently showed that AML from various cytogenetic risk groups, healthy bone marrow and CD34^+^ cells, could be distinguished based on protein expression and mass spectrometry (MS) peak patterns corresponding to post-translational modification differences.

2-DE has been used to analyse CD133^+^ progenitor cell fractions from patients with various leukemic disorders. Ten potential biomarkers were identified which included nuclear protein associated with mitotic apparatus (NuMA), heat shock proteins and redox regulators (Ota et al. 2003). In addition, Cui et al. (Cui et al. 2005) used 2-DE to profile samples from acute leukemia patients and differentially expressed spots were identified by MS. They found distinct protein profiles in FAB subtypes (M2, M3 and M5). Matrix-assisted laser desorption and ionization time-of-flight (MALDI-TOF) MS and its derivative surface enhanced laser desorption and ionization (SELDI)-TOF MS are enabling technologies which have increased the sensitivity and throughput for the detection of novel leukemia antigens. MALDI-TOF MS has previously been applied to chronic myeloid leukemia (Knights et al. 2006) to identify processed and presented epitopes eluted from MHC which led to the identification of a novel peptide from PRTN3, which was detected among the more abundant MHC-ligands. It can only be a matter of time before the same technology is used to identify epitopes in LAAs that are processed and presented on primary AML samples.

## Future Directions

In the future it would be hoped that microarray could be used to personalize therapy, identify patients who will develop drug resistance, those who will respond well to novel therapies and those who are likely to survive conventional treatment. These studies demonstrate the potential of 2-DE and MS to molecularly characterize acute leukemia providing further insights into leukemogenesis, defining subgroups and promoting the identification of new targets for specific treatment approaches.

Proteomic techniques such as differential in-gel electrophoresis (DIGE), multidimensional protein-identification technology (MudPIT) and field effect transistor (FET)-based protein detection are being applied to the identification of cellular and serum biomarkers (reviewed in Ludwig and Weinstein, 2005). Such studies are in their infancy but have the potential to identify proteins and post-translationally modified forms of proteins associated with cancers, which may include some novel targets for immunotherapy.

Down regulation of eukaryotic initiation factor 5A has been recently described as a novel target for hypusination inhibitors (HI) and, furthermore, the combination of Imatinib and HI produced a synergistic effect that was restricted to Imatinib resistant BCR-ABL^+^ cells (Balabanov et al. 2006). The identification of mechanistic markers for AML could also provide a new avenue to elucidate potential immunotherapeutic targets. BAFF and APRIL have been shown to be abnormally expressed on the membrane of B-cell chronic lymphocytic leukemia (B-CLL) blasts thereby conferring resistance to apoptosis (Kern et al. 2004). In fact, a soluble form of BAFF was also detected in patient sera but not in healthy donor controls.

SNPs analysis provides a rapid way in which regions of DNA within the genome with an abnormal gain or loss can be identified, enabling the detection of oncogenes and/or tumor suppressor genes, respectively, which are involved in the pathogenesis of a particular disease. Fitzgibbon et al. (Fitzgibbon et al. 2005) used SNPs analysis to show that approximately 20% of AML patients exhibit large-scale cryptic regions of acquired homozygosity in the form of uniparental disomy (UPD). In 7 of 13 of these patients they found concurrent homozygous mutations at four distinct loci (WT1, FLT3, CEBPA and RUNX1). UPD has also been identified as the cause for the cryptic chromosomal aberration for the inactivation of the NF1 gene, a tumor suppressor gene, in juvenile myelomonocytic leukemia (Stephens et al. 2006). MicroRNAs are a large family of highly conserved non-coding genes thought to be involved in temporal and tissue-specific gene regulation. Calin et al. (Calin et al. 2002) showed that in B-CLL, the microRNA cluster mir15a-mir16 was located in a region deleted or mutated in more than 68% of patients. These data suggest that SNPs and microRNAs have the potential to help elucidate important regulatory pathways in AML, which may help identify biomarkers of AML and targets for immunotherapy.

## Concluding Remarks

We have described the expanding field of AML antigen identification and our increasing understanding of the role these gene products play in leukemogenesis. We detail the dual role that some of these LAAs play as biomarkers of prognosis, MRD and survival while a number of preceding reviews have described in detail the role of these antigens as potential targets for the immunotherapy of AML. It remains to be determined whether these antigens will be predictive of disease progression, response to treatment and whether they can play a similar role in predicting outcome to treatment in the same way that cytogenetic findings do. Immunotherapies continue to be developed to target these LAAs as treatments for AML and our growing understanding of the role LAAs play in the pathogenesis of leukemia can only aid in the development of effective therapies.

## Abbreviations

AML: acute myeloid leukemia
APL: acute promyelocytic leukemia
ATRA: all-trans-retinoic acid
B-CLL: B-cell chronic lymphocytic leukemia
CT: cancer-testis
CTL: cytotoxic T-lymphocyte
FAB: French-American-British
HI: hypusination inhibitors
HSP: heat shock protein
ITD: internal tandem duplication
LAA: leukemia associated antigen
MDS: myelodysplastic syndrome
MGEA6: meningioma antigen 6
MPD: myeloproliferative disease
MS: mass spectrometry
NK: natural killer
PRAME: preferentially expressed antigen of melanoma
PRTN3: proteinase 3
RAGE-1: renal antigen 1
RHAMM: receptor for hyaluronic acid-mediated motility
RQ-PCR: real-time PCR
SAGE: serial analysis of gene expression
SCT: stem cell transplant
SEREX: serological analysis of recombinant cDNA expression libraries
SNPs: single nucleotide polymorphisms
UPD: uniparental disomy
WHO: World Health Organization
WT1: Wilm’s Tumor gene 1
